# Chylothorax and Chylopericardium: A Complication of Long-Term Central Venous Catheter Use

**DOI:** 10.1155/2019/4908259

**Published:** 2019-07-11

**Authors:** James Livesay, Isaac Biney, J. Francis Turner

**Affiliations:** Department of Medicine, University of Tennessee Graduate School of Medicine, Knoxville, TN, USA

## Abstract

The development of chylothorax and chylopericardium is an uncommon complication of the long-term use of central venous catheters. We describe a unique case of an end stage renal disease patient on hemodialysis with a left jugular tunneled catheter who developed superior vena cava syndrome. Our patient presented with both a large pleural and pericardial effusion that despite drainage continued to reaccumulate. Further imaging with CT scan of the thorax revealed stenosis of the superior vena cava leading to recurrent chylothorax and chylopericardium.

## 1. Introduction

Chylothorax and chylopericardium refer to the accumulation of chyle in the pleural and pericardial space, respectively. Chyle is a milky-appearing fluid containing high levels of fat, proteins, and immunoglobulins, which is carried back to the venous system from the cisterna chyli via the thoracic duct [1]. The thoracic duct originates from the cisterna chyli and terminates at the junction of the left subclavian and internal jugular veins. The volume of chyle ranges from 10 to 100 mL/kg body weight, in a normal adult, impacted by factors including diet, intestinal absorption, and degree of physical activity [2].

Both chylothorax and chylopericardium can be a direct result of superior vena cava (SVC) syndrome, which is best described as obstruction of the SVC interrupting normal venous return of blood from the head, upper extremities, and thorax to the right atrium [3]. Causes of SVC syndrome include direct trauma, surgical injury to the thoracic duct, malignancy, or as in this case central venous lines [4]. SVC syndrome secondary to central venous lines is typically a direct result of either thrombosis or vessel wall thickening leading to stenosis. Many cases present as a result of thrombosis around the catheter leading to SVC obstruction requiring removal of the catheter and long-term anticoagulation. The other common cause of SVC syndrome is SVC stenosis best defined as a diameter reduction >50% with or without upstream collaterals [5]. We report a case of SVC stenosis in an end stage renal disease (ESRD) patient on hemodialysis with a chronically indwelling left jugular central catheter leading to both chylothorax and chylopericardium.

## 2. Case Presentation

A 45-year-old male was admitted to the hospital with a one-day history of abdominal pain and dyspnea. The abdominal pain started the day of admission; however, he noted worsening shortness of breath and a productive cough with clear sputum over the course of several days. He also noted a 20-pound weight loss occurring over a three-month period but denied fevers, chills, or night sweats. Physical exam was notable for tachycardia, distant heart sounds, and decreased breath sounds of the lung bases bilaterally. Vitals at presentation included a temperature of 99.2°F, heart rate of 104, respiratory rate of 20, blood pressure 182/106 mmHg, and an oxygen saturation of 95% of 2 liters via nasal cannula.

Our patient has a past medical history significant for hypertension, chronic anemia, and end stage renal disease, due to focal segmental glomerular nephritis, on hemodialysis three times per week. He has required hemodialysis for approximately four years and unfortunately does not have an arteriovenous fistula due to financial restraints; therefore, his dialysis access is via a left jugular tunneled catheter. He has required multiple tunneled catheters over the past four years.

**Figure 1:**
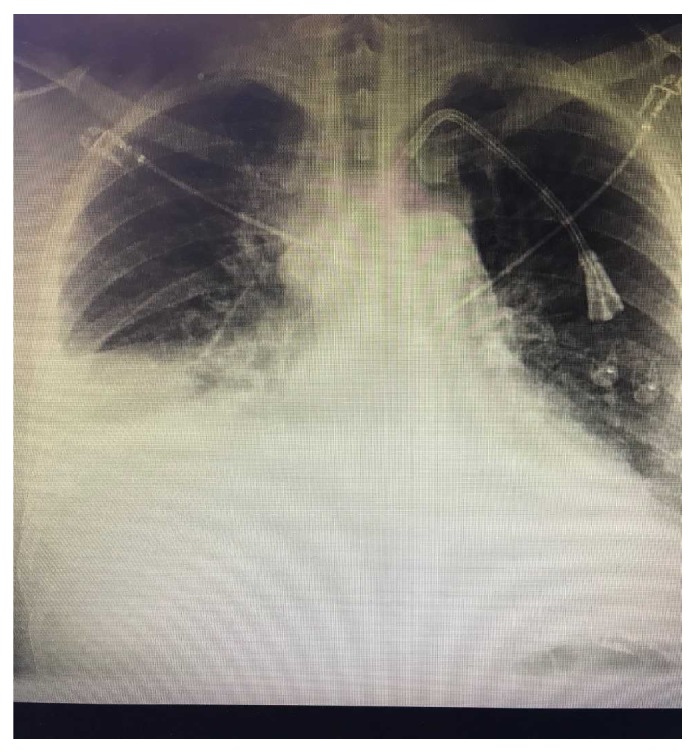
Initial chest x-ray demonstrating pleural effusion and abnormal cardiac silhouette.

Initial metabolic panel was within normal limits except for his creatinine of 7.99 mg/dL. Complete blood cell count revealed anemia with a hemoglobin of 10.6 g/dL and hematocrit of 33.7% but otherwise within normal limits. Chest x-ray was remarkable for bilateral pleural effusions and prominence of the cardiopericardial silhouette consistent with pericardial effusion (Figure 1). Given his abdominal pain a CT of the abdomen and pelvis without intravenous contrast was performed showing large right and small left pleural effusions and a large pericardial effusion. Since the CT scan was able to capture a majority of both the pleural and pericardial effusions a dedicated CT scan of the thorax was initially deferred.

Given his dyspnea and pleural effusions our patient underwent a right-sided thoracentesis by interventional radiology with removal of 1.5 liters of cloudy amber-colored fluid. Body fluid studies revealed a total protein of 3.3 g/dL, LDH 110, glucose 106, RBC 45, WBC 993, lymphocyte predominance of 91%, and pH of 7.0. Serum LDH was 247 and serum protein was of 6.6 g/dL. Using Light's Criteria (Table 1) our patient did not meet criteria for exudative effusion; however, results were borderline with a pleural fluid protein/serum protein ratio of 0.5 and pleural fluid LDH/serum LDH ratio of 0.445. Acid fast smear and cultures were obtained and resulted negative. Cytology was negative for malignancy but showed many small mature lymphocytes, mesothelial cells, and a few acute inflammatory cells.

Following thoracentesis, he had significant improvement in dyspnea and oxygen saturation improved to the upper 90s on room air. Regarding the pericardial effusion, a transthoracic echocardiogram was obtained revealing a large circumferential pericardial effusion measuring 1.67 cm anteriorly and 1.29 cm posteriorly. The chambers of the right atrium (RA) and right ventricle (RV) were very small, with complete obliteration of the RV chamber during systole, suggesting high intrapericardial pressure. The IVC was collapsible, measuring 1.67 cm, with a 50% collapse during inspiration. No diastolic collapse of the RV or complete systolic collapse of the RA was noted. Cardiology was consulted and during their physical exam he displayed no jugular venous distention, negative Kusmaul's sign, and no clinical signs of tamponade. An EKG was obtained showing sinus rhythm and no electrical alternans; thus pericardiocentesis was deferred.

Over the next four days he continued his regularly scheduled hemodialysis sessions and showed improvement in the pericardial effusion. Give hemodynamic stability and significant improvement there were no plans for intervention and the decision was made to closely follow with cardiology as an outpatient. However, on the planned day of discharge he developed supraventricular tachycardia and was kept another night for observation and evaluation by electrophysiology. During this time a repeat chest X-ray was performed revealing a reoccurrence of his pleural effusions; therefore, pulmonary medicine was consulted to further investigate.

**Figure 2:**
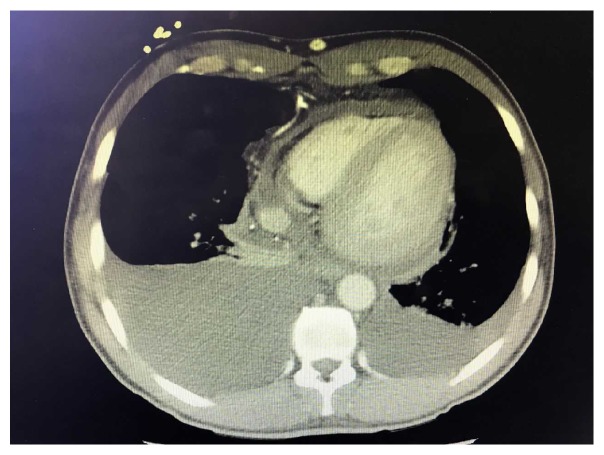
Demonstrates reaccumulation of pleural and pericardial fluid.

**Figure 3:**
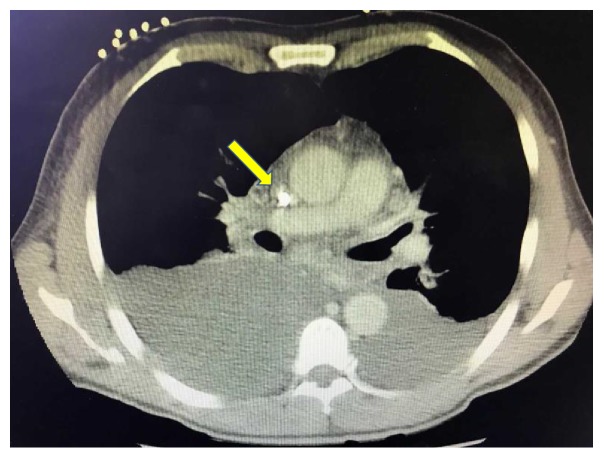
Demonstrates the stenotic area in the SVC around the catheter.

Previous body fluid studies were reviewed, and, given significantly elevated lymphocytes of 91% and borderline Light's Criteria for exudative effusion, there was initial concern for common etiologies including tuberculosis, sarcoidosis, and malignancy. Our patient immigrated from Mexico to the United States approximately 20 years ago but denied any Tuberculosis exposure or previous diagnosis. He did have a 20-pound weight loss but denied other B like symptoms. We elected to further investigate the thorax for the presences of anatomical abnormalities, so a CT thorax was obtained. CT demonstrated a reaccumulation of the right pleural effusion and persistent moderate to large pericardial effusion (Figure 2) but surprisingly showed stenosis or occlusion of the superior vena cava (Figure 3). This new finding was discussed with radiology that further commented on mediastinal collaterals and evidence of SVC stenosis particularly around the patient's tunneled catheter.

A repeat thoracentesis was performed and 1.6 liters of milky fluid with a pink tinge was removed. Body fluid was remarkable for total protein of 4.6 g/dL, glucose 72, triglycerides 1,056 mg/dL, cholesterol 116 mg/dL, RBC 423,195, WBC 1,760, lymphocytes 86%, and pH 8.0. These findings were consistent with chylothorax likely form newly discovered SVC stenosis.

Other labs to evaluate for autoimmune etiology including rheumatoid factor, c-ANCA, p-ANCA, ANA, and hepatitis screen were also obtained and were unremarkable.

**Figure 4:**
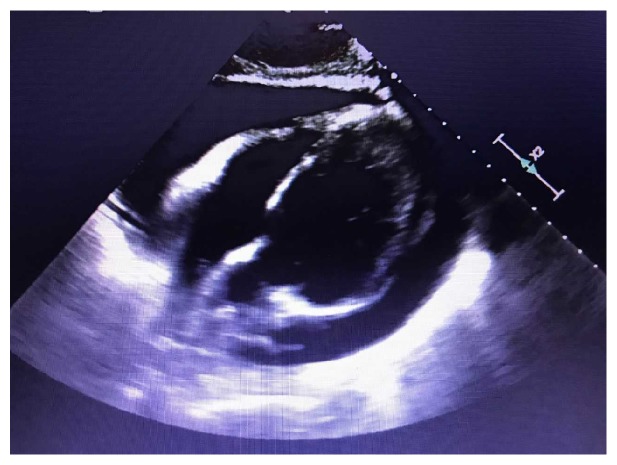
Echocardiogram prior to pericardial drain placement showing large pericardial effusion.

Repeat echocardiogram was performed and worsening pericardial effusion was noted revealing RA collapse and diastolic RV collapse suggestive of cardiac tamponade (Figure 4). Hemodynamically the patient remained stable and a scheduled pericardial drain was placed with aspiration of 775 mL of bloody fluid consistent with chylopericardium.

Given recurrent pleural and pericardial effusions vascular surgery was consulted for SVC stenosis. He underwent angioplasty of the SVC and left brachiocephalic vein using a 12x4 angioplasty balloon and replacement of the tunneled hemodialysis catheter into the left jugular vein. Ultimately, after balloon angioplasty, high triglyceride diet, and draining both the pleural and pericardial effusion his symptoms significantly improved, and he was discharged home. There are plans to establish permanent hemodialysis access by creating an AV fistula in the near future.

## 3. Discussion

The unique presentation of chylothorax and chylopericardium secondary to SVC stenosis, as seen in our patient, is exceptionally rare. A recent study by Labriola and colleagues showed SVC stenosis is not uncommon as once thought, but the clinical manifestations of SVC stenosis are infrequent in patients with absence of a peripheral vascular access. Their retrospective study found SVC stenosis occurs at a rate of 0.14 cases/1000 catheter days corresponding to 1.8 episodes per year in a center of 100 hemodialysis patients. However, the majority of SVC stenosis was diagnosed in the setting of catheter malfunction with only two cases diagnosed by the classical symptoms of SVC syndrome including facial and upper extremity edema [5]. While rare the development of chylothorax alone carries a mortality of 10% in most major medical centers [2]. Once central vein obstruction occurs it may quickly become clinically significant as the thoracic duct transports approximately 4 liters of chyle per day in the normal adult [2].

Regarding our patient the initial body fluid obtained during thoracentesis was not diagnostic of chylothorax or chylopericardium. Studies revealed a borderline exudative process, per Light's Criteria, and significantly elevated lymphocytes. Common etiologies to explain this presentation include malignancy, sarcoidosis, and tuberculosis. Further history elicited the fact that our patient had several risk factors that could explain his presentation including a 20-pound weight loss over the course of a few months and decreased appetite raising concern for malignancy. He immigrated from Mexico approximately 20 years ago raising concern for tuberculosis exposure; however, to the best of his knowledge he had not been exposed to or formally diagnosed with tuberculosis. Given his atypical presentation a literature search revealed an article reported by Alkayed and colleagues describing a 16-year-old-female with Li Fraumeni syndrome and a chronic dwelling tunneled catheter diagnosed with chylopericardium and chylothorax. Body fluid studies were similar to our patient including an elevated WBC with lymphocyte predominance [4].

The diagnosis was initially delayed as the body fluid was amber-colored and triglycerides were not obtained. As previously mentioned, the lymphocyte predominance did not fit his clinical picture, but after further imaging and research SVC syndrome moved high on the differential. Chylothorax and chylopericardium resulted from leakage of chyle into the chest. Chyle consists of not only fats and proteins but immunoglobulins, T-lymphocytes, and B-lymphocytes, which explains the significantly elevated lymphocyte count in our patient's fluid studies [2]. Given the loss of protein and fats it is imperative to consider nutrition in this patient population. Their diets should include high-content medium chain triglycerides as this will allow a bypass of the intestinal lymph system and absorption into the portal system [6].

CT thorax is crucial in these cases in not only diagnosing SVC syndrome but differentiating thrombus formation from stenosis. In this case, CT revealed central vein stenosis around the catheter with evidence of collateral veins. Repeat thoracentesis and a newly placed pericardial drain allowed us to retest his body fluid which was consistent with chylothorax and chylopericardium. Body fluid studies revealed significantly elevated triglycerides and lymphocytes along with a gross milky appearance.

In our patient SVC syndrome was a result of central vein stenosis and thrombus formation was ruled out. The underlying pathology of central vein stenosis is a direct result of chronic indwelling central vein catheters leading to intimal hyperplasia of smooth muscle cells and vein wall thickening [7]. Treatments of these complications vary from conservative with fluid drainage and nutrition support to thoracic duct surgery and pleurodesis. As performed in our patient balloon angioplasty is recommended for symptomatic patients and stenting for recurrent stenosis [1]. Talc pleurodesis, which has been well documented in malignant effusions, has shown benefit in this particular subset of patients; however, current literature lacks evidence in nonmalignant effusions [1].

## 4. Conclusion

In summary, patients with chronic indwelling catheters are at risk for SVC syndrome, including stenosis and thrombosis, ultimately leading to chylothorax and/or chylopericardium. As central venous catheter use increases these complications will likely become more common and can be difficult to diagnose. Therefore, clinicians should be aware of the complications associated with long-term central venous catheter especially when these patients present with dyspnea, pleural effusions, pericardial effusions, or upper extremity and facial edema.

## Figures and Tables

**Table 1:** Light's Criteria.

Pleural Fluid	PF/Serum Protein Ratio	PF/Serum LDH Ratio	PF LDH > Upper 2/3 Limit of Normal
Transudative	<0.5	<0.6	<2/3

Exudative	≥0.5	≥0.6	≥2/3
